# Perspectives on tracking data reuse across biodata resources

**DOI:** 10.1093/bioadv/vbae057

**Published:** 2024-04-25

**Authors:** Karen E Ross, Frederic B Bastian, Matt Buys, Charles E Cook, Peter D’Eustachio, Melissa Harrison, Henning Hermjakob, Donghui Li, Phillip Lord, Darren A Natale, Bjoern Peters, Paul W Sternberg, Andrew I Su, Matthew Thakur, Paul D Thomas, Alex Bateman, and the UniProt Consortium, Alex Bateman, Maria-Jesus Martin, Sandra Orchard, Michele Magrane, Shadab Ahmad, Emily H Bowler-Barnett, Hema Bye-A-Jee, Paul Denny, Tunca Dogan, ThankGod Ebenezer, Jun Fan, Leonardo Jose da Costa Gonzales, Abdulrahman Hussein, Alexandr Ignatchenko, Giuseppe Insana, Rizwan Ishtiaq, Vishal Joshi, Dushyanth Jyothi, Swaathi Kandasaamy, Antonia Lock, Aurelien Luciani, Jie Luo, Yvonne Lussi, Pedro Raposo, Daniel L Rice, Rabie Saidi, Rafael Santos, Elena Speretta, James Stephenson, Prabhat Totoo, Nidhi Tyagi, Preethi Vasudev, Kate Warner, Rossana Zaru, Supun Wijerathne, Khawaja Talal Ibrahim, Minjoon Kim, Juan Marin, Alan J Bridge, Lucila Aimo, Ghislaine Argoud-Puy, Andrea H Auchincloss, Kristian B Axelsen, Parit Bansal, Delphine Baratin, Teresa M Batista Neto, Jerven T Bolleman, Emmanuel Boutet, Lionel Breuza, Blanca Cabrera Gil, Cristina Casals-Casas, Elisabeth Coudert, Beatrice Cuche, Edouard de Castro, Anne Estreicher, Maria L Famiglietti, Marc Feuermann, Elisabeth Gasteiger, Sebastien Gehant, Arnaud Gos, Nadine Gruaz, Chantal Hulo, Nevila Hyka-Nouspikel, Florence Jungo, Arnaud Kerhornou, Philippe Le Mercier, Damien Lieberherr, Patrick Masson, Anne Morgat, Ivo Pedruzzi, Sandrine Pilbout, Lucille Pourcel, Sylvain Poux, Monica Pozzato, Manuela Pruess, Nicole Redaschi, Catherine Rivoire, Christian J A Sigrist, Shyamala Sundaram, Anastasia Sveshnikova, Cathy H Wu, Cecilia N Arighi, Chuming Chen, Yongxing Chen, Hongzhan Huang, Kati Laiho, Minna Lehvaslaiho, Peter McGarvey, Darren A Natale, Karen Ross, C R Vinayaka, Yuqi Wang, Jian Zhang

**Affiliations:** Protein Information Resource, Department of Biochemistry and Molecular & Cellular Biology, Georgetown University Medical Center, Washington, DC 20007, United States; Evolutionary Bioinformatics Group, SIB Swiss Institute of Bioinformatics, 1015 Lausanne, Switzerland; Department of Ecology and Evolution, University of Lausanne, 1015 Lausanne, Switzerland; DataCite, 30167 Hannover, Germany; Global Biodata Coalition, Strasbourg 67080, France; Department of Biochemistry & Molecular Pharmacology, NYU Grossman School of Medicine, New York, NY 10012, United States; Literature Services, European Molecular Biology Laboratory, European Bioinformatics Institute (EMBL-EBI), Wellcome Genome Campus, Hinxton CB10 1SD, United Kingdom; Molecular Systems, European Molecular Biology Laboratory, European Bioinformatics Institute (EMBL-EBI), Wellcome Genome Campus, Hinxton CB10 1SD, United Kingdom; Chan Zuckerberg Initiative, Redwood City, CA 94063, United States; School of Computing, Newcastle University, Newcastle upon Tyne NE4 5TG, United Kingdom; Protein Information Resource, Department of Biochemistry and Molecular & Cellular Biology, Georgetown University Medical Center, Washington, DC 20007, United States; Center for Vaccine Innovation, La Jolla Institute of Immunology, La Jolla, CA 92037, United States; Division of Biology and Biological Engineering, California Institute of Technology, Pasadena, CA 91125, United States; Department of Integrative Structural and Computational Biology, The Scripps Research Institute, La Jolla, CA 92037, United States; Data Services, European Molecular Biology Laboratory, European Bioinformatics Institute (EMBL-EBI), Wellcome Genome Campus, Hinxton CB10 1SA, United Kingdom; Department of Population and Public Health Sciences, University of Southern California, Los Angeles, CA 90089, United States; MSCB, European Molecular Biology Laboratory, European Bioinformatics Institute (EMBL-EBI), Wellcome Genome Campus, Hinxton CB10 1SD, United Kingdom

## Abstract

**Motivation:**

Data reuse is a common and vital practice in molecular biology and enables the knowledge gathered over recent decades to drive discovery and innovation in the life sciences. Much of this knowledge has been collated into molecular biology databases, such as UniProtKB, and these resources derive enormous value from sharing data among themselves. However, quantifying and documenting this kind of data reuse remains a challenge.

**Results:**

The article reports on a one-day virtual workshop hosted by the UniProt Consortium in March 2023, attended by representatives from biodata resources, experts in data management, and NIH program managers. Workshop discussions focused on strategies for tracking data reuse, best practices for reusing data, and the challenges associated with data reuse and tracking. Surveys and discussions showed that data reuse is widespread, but critical information for reproducibility is sometimes lacking. Challenges include costs of tracking data reuse, tensions between tracking data and open sharing, restrictive licenses, and difficulties in tracking commercial data use. Recommendations that emerged from the discussion include: development of standardized formats for documenting data reuse, education about the obstacles posed by restrictive licenses, and continued recognition by funding agencies that data management is a critical activity that requires dedicated resources.

**Availability and implementation:**

Summaries of survey results are available at: https://docs.google.com/forms/d/1j-VU2ifEKb9C-sW6l3ATB79dgHdRk5v_lESv2hawnso/viewanalytics (survey of data providers) and https://docs.google.com/forms/d/18WbJFutUd7qiZoEzbOytFYXSfWFT61hVce0vjvIwIjk/viewanalytics (survey of users).

## 1 Introduction

Data reuse among biodata resources is ubiquitous. For example, UniProt (UniProt Consortium 2023) provides data to many other resources, including protein resources that use UniProt sequence data and resources such as the Gene Ontology (GO) (Gene Ontology Consortium 2019), which collects UniProt GO annotations and then distributes them to many downstream users. Conversely, UniProt distributes the Gene Ontology and annotations produced by other GO Consortium members and is a major consumer of protein related data from numerous other data resources. Free and open data use and reuse is an important philosophy that predominates in the molecular biology field. However, data reuse is very difficult to document and quantify, limiting our ability to assess the impact of biodata resources.

It is important to note the distinction between data use by end users and data reuse among resources. While there is still work to be done to fully understand the impact of biodata resources on the work of end users, a variety of strategies to help close this gap are being implemented. These include use of web analytics, better education of researchers on good data citation practices, encouraging publishers to establish higher standards for data citation, and literature mining. For example, the Chan Zuckerberg Initiative (CZI) is mining mentions of software from full-text articles (Istrate *et al.* 2022) and is extending this approach to datasets (Imker *et al.* 2023). In another example, Europe PMC is tracking citations of the Elixir Core Data Resources in the literature using three strategies: (i) detecting mentions of the resource name; (ii) detecting mentions of resource unique identifiers; and (iii) detecting citations of other articles that describe the resource in detail (Drysdale *et al.* 2020).

Tracking data reuse among resources is a less well-studied and much more challenging problem (Bell and Lord 2017). EMBL-EBI has tracked such reuse among its own resources and between its own resources and other data resources (Cook *et al.* 2016, Cook *et al.* 2020). Similarly, the ELIXIR Core Data Resources have also tracked data exchanged among the Core Data Resources themselves (Drysdale *et al.* 2020). Collecting data for these studies is challenging: information is usually exchanged programmatically, through code written over many years, with no formal tracking. Consequently, collecting the data requires review of each resource’s code base to identify data exchanges. An additional difficulty is that for open data resources it is only possible to tally data that are brought in from another resource; for “outgoing” data it is often not possible to distinguish between reuse by other resources and use by end users. Moreover, resources sometimes obtain data from other resources that are themselves reusing the data, creating chains of reuse that can be several times removed from the original source. This indirect data use may represent a significant fraction of overall usage; however, tracking it would require redistributors, who typically integrate data from multiple sources, to separately track and report usage statistics for each redistributed dataset.

Understanding the full extent of data reuse is important. First, funding for data resources is limited, and accurate measurement of the usage of data from each resource would be helpful to grant agencies when prioritizing funding decisions. In addition, source databases make regular updates that improve and expand their data. These updates are critical for end users: for instance, data analyses carried out with newer versions of the GO and Reactome knowledgebases have been shown to yield improved biological insights (Wadi *et al.* 2016). If resources were aware of everyone who was using their data, they would be in a better position to help downstream resources synchronize with new data releases.

To define the challenges surrounding data reuse and to devise recommendations for more effective and transparent data sharing among resources, the UniProt Consortium hosted a one-day virtual workshop in March 2023. It was attended by ∼35 people, including representatives from biodata resources and scientific publishing, experts in data management, and NIH program managers. Workshop discussions focused on three areas: (i) strategies for tracking data reuse; (ii) best practices for reusing data; and (iii) challenges in reusing data and tracking reuse. Prior to the workshop, we distributed two surveys, one for data resource providers and one for users. Results were shared with the participants and helped inform discussions.

## 2 The current landscape of data reuse among biodata resources

**Figure 1.**
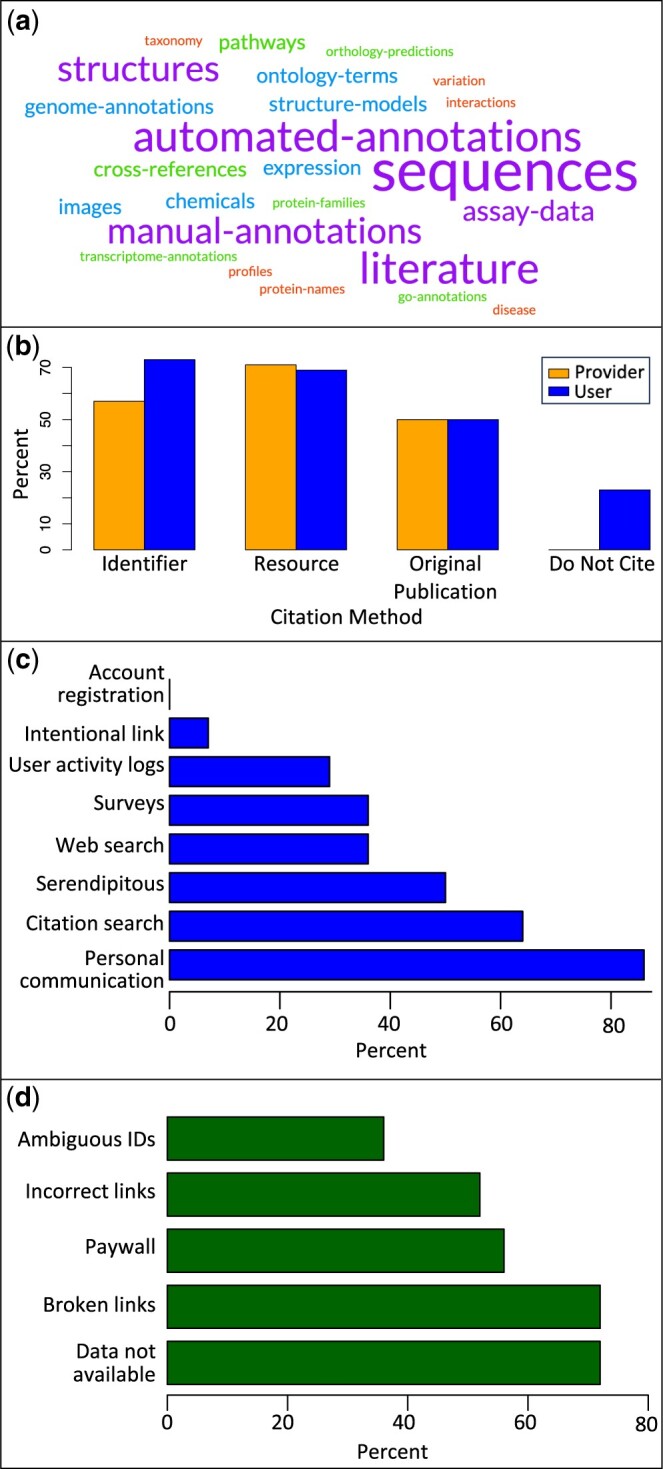
Selected survey responses. (a) Word cloud showing data types that providers reported reusing. Larger words were mentioned more frequently. Created with Free Word Cloud Generator (https://www.freewordcloudgenerator.com/). (b) Frequency of citation methods for reused data as reported by providers (left bar of each pair) and users (right bar of each pair). (c) Frequency of methods for discovering/tracking data reuse as reported by providers. (d) Frequency of challenges accessing the original source of reused data as reported by users. Full survey results: Providers: https://docs.google.com/forms/d/1j-VU2ifEKb9CsW6l3ATB79dgHdRk5v_lESv2hawnso/viewanalytics; Supplementary File 1 Users: https://docs.google.com/forms/d/18WbJFutUd7qiZoEzbOytFYXSf WFT61hVce0vjvIwIjk/viewanalytics; Supplementary File 2.

We carried out two surveys prior to the workshop, one aimed at data resource providers and one aimed at users of data resources (Supplementary Files 1 and 2). The surveys were advertised via the UniProt home page and social media, as well as via professional societies, including the International Society for Biocuration and the International Society for Computational Biology during February 2023; additionally, responses were solicited from workshop invitees via email. According to our small survey of resource providers (14 responses), data reuse among resources is widespread; all respondents indicated that their resource reused data, in most cases using more than five other resources, and all respondents considered the reused data to be very important to their work (score of 4–5 on a 5-point Likert scale). A wide variety of data is reused, with sequences, structures, annotations, and literature citations among the most common data types mentioned (Fig. 1a). Resources do often obtain data via mechanisms designed for data sharing such as programmatic interfaces (e.g. APIs) and bulk downloads from website repositories or FTP sites. However, informal mechanisms of data sharing such as personal communication with resource developers and even manual copying from a resource’s public interface were also cited.

In our survey, all data providers indicated that they cite reused data using methods such as using identifiers minted by the source database, citing the source by name, or citing the publication where the data was originally reported. However, 23% of respondents to our user survey reported that not all resources provide citations to the resources they reuse (Fig. 1b). This indicates that good citation practices are not universal among resources, and raises the possibility that even when resources provide citations, they are overlooked or misinterpreted by users. Citations may also lack information, such as version numbers, that is critical for reproducibility.

All data provider respondents were also aware that other resources were using their data. The most common way that respondents learned about reuse was through personal communication with the other resource (Fig. 1c). Respondents also noted active efforts to identify reuse such as web searches, citation searches, and surveys. Only about a quarter of respondents (28%) discovered reuse through automated means such as monitoring user activity logs. Interestingly, half of respondents indicated that they had discovered only serendipitously that their data was being reused. Most providers rated their ability to track reuse of their data as moderate to good.

According to our small user survey (26 responses), awareness of data reuse among resources is fairly high, with three-quarters of users responding that many or all of the resources that they are familiar with reuse data. More than half of users reported that they sometimes had difficulty accessing the original source of reused data due to issues such as broken or incorrect web links, lack of data availability, or paywalls (Fig. 1d). These results suggest that there is room for improvement in linking shared data among resources.

## 3 Challenges

Our surveys and discussions at the workshop highlighted several challenges faced by data providers in tracking reuse of their own data as well as responsibly reusing data from other resources.

### 3.1 Tracking data reuse has a cost

According to the survey of data providers, over half (57%) devote time, personnel, and/or other resources to monitoring data reuse. However, funding for improving data tracking is lacking. Efforts by independent organizations to track reuse across multiple resources, such as the network of data exchange among resources at the European Bioinformatics Institute (Cook *et al.* 2020), requires an investment not only by the organization conducting the study, but also by the individual data resources involved, who must compile the necessary information, often manually. These efforts detract from the primary mission of the resources.

Data providers can facilitate tracking by associating their data with persistent identifiers such as DOIs, which have gained significant traction within the publishing community (Cousijn *et al.* 2018). The DOI infrastructure has invested in collection and storage of metadata associated with DOI registration, such as persistent identifiers for people (ORCIDs), institutions (RORIDs) and funders and grants, as well as crosslinking between different outputs using DOIs. However, there is a direct cost associated with registration of DOIs with agencies such as Crossref and DataCite that can burden smaller, less well-funded resources as well as resources that generate very high numbers of records. For instance, the number of accession numbers generated for European Nucleotide Archive records alone (3.8 billion accessions) exceeds the total number of DOIs registered with Crossref (164.24 million DOIs). The metadata associated with DOIs, while useful for linking and tracking reuse, is also costly for data providers to assemble.

Accession numbers are well established persistent identifiers within the biological database community (Cousijn *et al.* 2018). They are low cost because, unlike DOIs, there is no direct cost to generate them. Services such as http://identifiers.org and https://n2t.net/ have been established to resolve accession numbers although currently the infrastructure does not extend to associated metadata. Europe PMC tracks accession citations in text for over 50 resources from EMBL-EBI and ELIXIR core databases (Drysdale *et al.* 2020). Over a 10-year period, 2013–23, more than 1.1 million citations to accession numbers have been identified in the text of open access content indexed in Europe PMC and resolved using identifiers.org. These citations are available via the front end Europe PMC website, as well as the Annotations API for programmatic users.

### 3.2 Tension between tracking reuse and open data

On the one hand, data providers have a legitimate interest in tracking how their data is used and reused. On the other hand, strategies that effectively track usage can introduce burdens that can hinder data access and reuse.

For example, requiring users of a resource to register makes it easier to track data use, but also deters users, introducing barriers to open sharing of data. A possible compromise would be to require registration only for bulk downloads of data or API access. According to our survey, these were the two most common methods by which resource providers obtained data from other resources, with 85% of respondents indicating they obtained data through bulk downloads and >90% indicating that they used APIs. Registration is less likely to be viewed as an obstacle by large-scale users, and in keeping with this, the Global Biodata Coalition (https://globalbiodata.org/) does not consider registration requirements for large-scale data access to be incompatible with open data policies for its Global Core Biodata Resources. Although a limited registration scheme appears to be a promising strategy for tracking data reuse, it is currently not widely implemented. Of the resource providers who responded to our survey, almost all permitted large-scale data access such as bulk downloads, but none required registration.

Licenses are sometimes used so that providers can maintain control over how their data is distributed. In other cases, resources use licenses for scientific/technical reasons; e.g., in order to support the use of stable identifiers in ontologies, the Protein Ontology (Natale *et al.* 2017) and other members of the Open Biomedical Ontologies (OBO) Foundry use a Creative Commons CC BY (attribution required) license to prevent downstream users from minting new identifiers for its terms. In still other cases, restrictive licenses may be required by organizations in order to protect intellectual property. However, this practice severely hampers data integration. Moreover, any non-open licensing of content is contrary to the spirit of open access and would preclude selection of the resource to become a GCBR or ELIXIR core data resource. Forgoing a license entirely is not an optimal solution because there are jurisdictions, such as the European Union, where it is not permissible to reuse a resource unless it has a license of some kind. The Creative Commons CC0 (Public Domain) designation is the most conducive to data sharing. Proponents of CC0 argue that tax-payer funded research should be in the public domain and that CC0 should be required by funders. There is also a role for education—when resource providers are informed about the limitations caused by restrictive licenses, they are often willing to change their practices.

### 3.3 Knowing that data is being used is not the same as knowing what it is used for

Detecting users who access large amounts of data either through analytics or registration does not reveal what happens to the data afterward. It does not distinguish between users who download data for one-time use from those who intend to widely redistribute the data via their own resource.

### 3.4 Data reuse by commercial entities is often untrackable

Commercial entities can be major reusers of data, aggregating data from many public resources in databases for internal use that may have large user groups. They often do not cite this data publicly in order to protect proprietary information from competitors. Public data may also be incorporated into commercial products. While this data may be cited within the product, the citations are not public and are therefore challenging to track. While establishing separate license requirements or terms of use for commercial entities is a possible solution, it is contrary to the letter and spirit of open access and is complicated and expensive to manage. Introducing procedures to verify whether a user is commercial or non-commercial would create friction for both website users and automated (API-based) data exchange. Thus, the community needs to consider alternative ways to encourage commercial users to acknowledge use of public resources.

### 3.5 Clearly citing source data also has a cost

Failure to accurately cite sources usually does not stem from an intention to hide this information; instead, accurately reporting provenance is labor-intensive, sometimes requiring extensive curation effort. The necessary investment of time and money can pose a challenge for even the best-funded resources and may be prohibitive for smaller resources.

Part of the problem is that current standards for data citation were modeled after literature citation practices. The publishing community has made efforts to embed data citation into its workflows and encourage authors to cite data within reference lists and data availability statements. There is also guidance to publishers on how to codify this in their mark up (https://jats4r.niso.org/data-citations/ and https://jats4r.niso.org/data-availability-statements/). However, this guidance is heavily influenced by citations practices for journal articles and books, and authors can be met with resistance if their datasets do not have established publishing identifiers, such as DOIs. These standards are not always a good fit for the ways that data is used and reused, but they are difficult to change because they are part of the scientific culture—i.e., they are the way people expect to get credit for their work.

At some point, after data has been redistributed multiple times, it becomes impractical to keep track of the entire chain of citations. For example, knowledge graphs (KG) are a powerful tool for integrating data from many sources, but it is complicated to exhaustively cite all of the sources that contribute to a KG.

Use of large language models (LLMs, e.g. ChatGPT) is likely to further disrupt traditional citation and data provenance practices. Currently, ChatGPT does not provide accurate citations for the information it provides and while the outright hallucination by LLMs that is common today will likely diminish in the near future, it is unlikely that LLMs will provide detailed information provenance. It will be important to establish criteria for when data no longer needs to be cited and is regarded as background knowledge.

### 3.6 The data on which resources rely is highly dynamic

Data resources are constantly being augmented and improved. While providing accurate, up-to-date data ultimately benefits science as a whole, it creates great challenges for resources that integrate data from multiple sources. Keeping track of when data sources have been updated and managing all of the downstream effects of an update is laborious, unrewarding work that is often not recognized as productive effort. Because data providers are usually unaware of how their data is being reused, they are unable to communicate effectively about updates or implement them in a way that minimizes disruption to downstream users.

### 3.7 Reporting the quality of the underlying data sources on which information is based is challenging

For the most part, all information provided by biodata resources is regarded as being of equal quality, even though this may not be a well-founded assumption. Some resources, such as UniProt, address this issue by labeling their data with Evidence & Conclusion Ontology (ECO) codes (Nadendla *et al.* 2022). While these codes provide some information about the data source, they are not designed to be confidence scores; data of varying quality can all be tagged with the same ECO code. Efforts to develop a confidence ontology have so far been unsuccessful because each resource has its own definition of confidence (Bastian *et al.* 2015). Developers of the Simple Standard for Sharing Ontological Mappings (SSSOM) consider confidence to be a desirable part of a mapping standard (Matentzoglu *et al.* 2022). The SSSOM provides a confidence field, so that users can filter mappings according to the degree of confidence needed for their use case. Contributions to a confidence score could include the quality of the method used to collect the data, the number of independent pieces of evidence supporting the data, and/or user rating of the validity of the data (https://github.com/mapping-commons/sssom/issues/338). Another approach is leave confidence assessment to the user by providing links to the raw data and describing that raw data using ontologies such as the Ontology of Biomedical Investigations (OBI) (Bandrowski *et al.* 2016). Ultimately, the curators of a resource are its gatekeepers; they determine which information and which source databases are sufficiently trusted to be included.

## 4 Principles

A number of guiding principles to address some of the challenges associated with data reuse emerged from our discussions.

### 4.1 Responsible reuse of data and support of data reuse

We encourage the community to develop a set of best practices for data reuse among resources. These practices could include: citation of each data element or dataset that is redistributed from another source, tracking and reporting of usage statistics (publicly and/or back to the data resource from which the data were obtained) for each redistributed data element or dataset, and an agreement to not redistribute “stale data”: to either update the reused data or retire the use of a data version entirely after a certain time.

First and foremost, resources should make it obvious from which other resources they are pulling data. Resources could be encouraged to list their “inputs” (data resources they use), ideally in a standardized, machine-readable format that is actively maintained as source database versions are updated. Just as software projects implement systems to automatically keep track of the code they are dependent on and make updates when necessary, databases could periodically run automated checks of their source databases to ensure they are using the latest versions. If this reporting is widely adopted, it could be used to build a network of resource interconnections, similar to the network built for European Bioinformatics Institute resources (Cook *et al.* 2016, Cook *et al.* 2020) and for the ELIXIR Core Data Resources (Drysdale *et al.* 2020).

Similarly, databases could list their “outputs” (resources that use their data) in a standardized format. For example, the Reactome biological pathway resource (Milacic *et al.* 2024) lists its external partners visibly on its webpage, which benefits all parties: Reactome can show that it is useful to others, and its partners get positive exposure. Being able to point to a list of resources that would lose critical functionality if their resource ceased to exist could be a powerful argument that data providers could make to funding agencies for continued support. Pages listing such partnerships can also spark ideas for new collaborations.

In addition to transparent documentation of inputs and outputs, resources reusing data should consider using a fine-grained citation scheme in which they associate each piece of data with the source database or publication. For example, this has been the longstanding policy for annotations in the Gene Ontology and UniProt. Fine-grained citation makes it possible to keep track of the cross-references to a piece of data, providing insight into inter-resource use of data.

Developing a standardized way to report confidence in data reused from other resources has been very challenging. Curator judgment will continue to be very important for deciding what information should be included in a resource. In some cases, the type of evidence for a piece of data relates to the confidence level; e.g., Gene Ontology annotations deriving from high-throughput experiments, which often have substantial false-positive rates, are flagged with specific evidence codes, which allows them to be identified and filtered if desired. It is also very important to provide links to the raw data whenever possible and avoid entangling the data and the current interpretation of the data, as the latter may change over time.

There was a consensus that restrictive licenses hamper data integration and that resources should avoid using licenses as a means to track data distribution. A Creative Commons CC0 designation a good choice for encouraging data sharing.

Finally, acknowledging the costs of careful data citation as well as the impracticality of accurately citing data that has been reused multiple times, it would be useful to establish criteria for when data no longer needs to be cited and is regarded as background knowledge.

#### 4.1.1 The alliance of genome resources data portal

The Alliance of Genome Resources, a consortium of the model-organism centric knowledgebases (MODs), has been engaged in a highly thoughtful and effective effort to make data from disparate model-organism databases available through a single web portal, and their experience can serve as a guide for other data reuse efforts. MODs encounter longstanding and difficult issues related to data accessibility and reuse. They contain uniquely deep and reliable annotations of the molecular biology of key model organisms and use similar data types—gene function, gene expression, genetic variation, phenotype, and human disease associations—to represent this knowledge. Historically, however, they differ in how these data types are modeled and displayed to end users. These differences impede development of common schemas and uniform user interfaces for data types across different organisms and represent a substantial inefficiency both for knowledgebases that must develop and maintain their own bioinformatic infrastructures and for users who must master them all and construct tools for data access and integration across resources. In a process that may provide a useful standard for the kinds of data harmonization efforts proposed here, the Alliance for Genome Resources is developing a common schema that builds on widely used community resources like the Gene Ontology and UniProt to develop strategies for annotating key molecular genetic data types. For example, all of the model organisms currently in the Alliance consortium have a concept of a transgene, but not a common representation of this concept. To implement a common data model and unified display for transgenes, a novel harmonized data model represents transgenes by two separate concepts—the transgene construct and the transgene allele—and defines relationships between the concepts. Specifically, a transgene construct is the DNA used to create a transgenic allele, and has explicit relationships to genes, gene segments, and to the transgenic alleles created using the construct. The transgenic allele represents a construct in the context of a genome. Transgenic alleles have relationships to constructs and genomes. Because the transgene data type is harmonized, data from all of the model organism-specific Knowledge Centers can be represented uniformly on the Alliance web portal (Bult and Sternberg 2023). While the goal is to harmonize model organism data and present it to the user in as seamless a fashion as possible, the Alliance is also mindful of the importance of preserving the provenance of the data. Therefore, they are developing a database that includes granular attribution that tracks the primary source and the chain of data reuse. For example, data from RefSeq via the Rat Genome Database (RGD) is sourced to NCBI as the primary source and RGD as a secondary source. In addition, data objects displayed in the Alliance portal often have IDs that are minted at the MODs. Still, achieving the optimal balance between data integration and faithful attribution is a work in progress. The project is in a rapid development phase to harmonize knowledge, store it, analyze it, and present it to the community through a web portal, direct downloads, and APIs (Alliance of Genome Resources Consortium 2023).

### 4.2 Tracking and assessing impact of data reuse among resources

It is important to develop standardized methods for tracking data reuse. Toward that goal, DataCite has developed the Code of Practice for Research Data Usage Metrics (https://www.projectcounter.org/counter-code-practice-research-data-usage-metrics-release-1/), which includes an open source script for tracking data use that can be easily implemented by data resources. Although it does not distinguish between users who obtain data for one-time use from those who intend to redistribute data, the tool is an important step toward making metrics more uniform and interoperable. To increase the reach of standardized metrics, data providers could request use of such tools by platforms that redistribute their data.

As a simple, low-effort method of capturing large-scale data use, resources could consider requesting voluntary registration for large-scale data access (e.g., bulk downloads and API access). While registration clearly can deter casual users, the consensus was that it would be less of a barrier to large-scale users such as other biodata resources. Use of a single credential for registration across resources would spare users from the need to keep track of different credentials for each resource they use. ORCIDs are increasingly being adopted as user identifiers across platforms in the biomedical domain; however, they are not ideal to track date reuse by resources because they are individual identifiers. Providers are likely to be more interested in the organization that wants their data rather than in the individual charged with executing the download. Conversely, individuals may not want to associate their personal ORCID with a task performed on behalf of their organization. Finally, although use of ORCIDs as a registration credential would not, *a priori*, be a violation of user privacy as long as the user explicitly opted-in to having their information stored, the resource storing the information would have an obligation to protect it from accidental release.

While there is an instinct to reach for technical solutions to document data reuse among resources, a lot could be learned about the value of data reuse from in depth studies of individual major use cases. For example, through discussions with bulk data users at the UK National Health Service Genomics Medicine Service and Genomics England, EMBL-EBI learned that thousands of patients each month are now receiving care-improving sequencing results. The data users confirmed that these results rely on bulk use of EMBL-EBI genomic reference data, the MANE gene annotation standards developed in collaboration by EMBL-EBI and NCBI, and the gene Variant Effect Predictor service of EMBL-EBI co-hosted resource Ensembl. This qualitative exploration of key user behavior revealed much more about impact than would have been apparent from analytics (where the entire UK National Health Service might appear as a single IP address).

### 4.3 How funding agencies can help

As a primary funder of many biodata resources, the NIH has a stake in ensuring that data is collected, curated, and shared as efficiently and transparently as possible. The NIH adopted the FAIR principles–Findability, Accessibility, Interoperability, and Reusability (Wilkinson *et al.* 2016)–for data stewardship in 2016 and has continued to build on those ideas.

The NIH 2023 Data Management and Sharing (DMS) Policy (https://sharing.nih.gov/data-management-and-sharing-policy) contains several provisions that may have a positive impact on data reuse. As of January 2023, all applicants for NIH funding must complete a detailed DMS plan, which includes sections on what data will be shared, how data will be distributed, what standards will be used, and what factors may limit data sharing and reuse. Additionally, budgets and budget justifications are expected to include costs associated with data sharing. The DMS plan provides an opportunity for resource developers to devise an optimal strategy for sharing data with both end users and other resources, and the budgeting requirements reflect that NIH regards responsible data sharing as worthy of monetary investment.

Because developers of AI/ML models are becoming increasingly important large-scale consumers of data, NIH has been soliciting administrative supplement applications since 2021 for projects that make NIH-supported data AI/ML ready. This funding mechanism allows biodata resources to guide how their data will be used and acknowledged in the AI/ML ecosystem where traditional citation practices may be impossible.

In the future, leadership from the NIH in future versions of the data management policy on the importance of less restrictive data licensing would be extremely helpful, especially in the context of AI/ML. Without the licenses that permit reuse, a large gap may develop between the potential biomedical impacts of AI and what is legally permissible.

## 5 Conclusions

In conclusion, data sharing and reuse are essential for the progress of biomedical science, and open science and open data practice are the foundation upon which the entire data ecosystem relies. Data deposited by research scientists, along with the knowledge in the research literature, have been organized and enhanced through the work of the thousands of biological data resources available today (Imker *et al.* 2023, Rigden and Fernández 2023). We can measure the impact of these resources in various ways, such as by citations or web statistics, but these measures only account for the end usage of the data and not for the vital and extensive sharing of data among resources in the biodata ecosystem.

The workshop described in this article highlights that while resource providers acknowledge the importance of citing reused data and the user awareness of data reuse by biodata resources is high, obstacles to clearly documenting the provenance of data persist. Conversely, the field is currently lacking the tools to measure reuse effectively, and understanding patterns of data reuse often requires detailed investigations of individual cases (Bell and Lord 2017).

The challenges surrounding data reuse are numerous: tracking and citing reused data entail resource-intensive efforts, and the tension between openness and sustainability poses a dilemma for data providers. Issues like restrictive licenses, untrackable reuse by commercial entities, and evolving citation practices, especially with the adoption of LLMs, further complicate responsible data sharing.

Several recommendations to address these challenges arose from workshop discussions: (i) development of a standardized machine-readable format for resources to document their inputs (resources they take data from) and their outputs (resources that use their data); (ii) adoption of fine-grained citation schemes that include the source of individual pieces of data and, when possible, the confidence in the underlying source; (iii) education to reduce the use of restrictive licenses that impede data sharing; (iv) requesting registration for bulk data downloads; (v) development of automated tools for tracking data reuse such as DataCite’s Code of Practice for Research Data Usage Metrics; and (vi) continued recognition by funding agencies that data management is a critical activity that requires an investment of resources.

The creation and adoption of simple, clear data standards will facilitate citation and tracking of reuse and ensure that credit is shared equitably among the biomedical knowledge infrastructure and that high quality data is easily accessible to the biomedical community.

## Supplementary Material

vbae057_Supplementary_Data

## Data Availability

The data underlying this article are available in the article and in its online supplementary material. Summaries of the survey results are available at: https://docs.google.com/forms/d/1j-VU2ifEKb9C-sW6l3ATB79dgHdRk5v_lESv2hawnso/viewanalytics (survey of data providers) and https://docs.google.com/forms/d/18WbJFutUd7qiZoEz-bOytFYXSfWFT61hVce0vjvIwIjk/viewanalytics (survey of users).
